# Remote Evidence-Based Programs for Health Promotion to Support Older Adults During the COVID-19 Pandemic and Beyond: Mixed Methods Outcome Evaluation

**DOI:** 10.2196/52069

**Published:** 2024-06-13

**Authors:** Lesley Steinman, Kelly Chadwick, Erica Chavez Santos, Sruthi Sravanam, Selisha Snowy Johnson, Elspeth Rensema, Caitlin Mayotte, Paige Denison, Kate Lorig

**Affiliations:** 1 Health Promotion Research Center, Department of Health Systems and Population Health, University of Washington School of Public Health Seattle, WA United States; 2 Office of Community Outreach and Engagement, Fred Hutch Cancer Center Seattle, WA United States; 3 Department of Anesthesiology and Pain Medicine, University of Washington School of Medicine Seattle, WA United States; 4 Sound Generations Seattle, WA United States; 5 Self Management Resource Center Aptos, CA United States

**Keywords:** older adults, health equity, rural, chronic disease, outcome evaluation, behavior change, technology, community based, evidence based, health promotion, mobile phone

## Abstract

**Background:**

Evidence-based programs (EBPs) for health promotion were developed to reach older adults where they live, work, pray, and play. When the COVID-19 pandemic placed a disproportionate burden on older adults living with chronic conditions and the community-based organizations that support them, these in-person programs shifted to remote delivery. While EBPs have demonstrated effectiveness when delivered in person, less is known about outcomes when delivered remotely.

**Objective:**

This study evaluated changes in remote EBP participants’ health and well-being in a national mixed methods outcome evaluation in January 1, 2021, to March 31, 2022.

**Methods:**

We used the RE-AIM (Reach, Effectiveness, Adoption, Implementation, and Maintenance) for equity framework to guide the evaluation. We purposively sampled for diverse remote EBP delivery modes and delivery organizations, staff, and traditionally underserved older adults, including people of color and rural dwellers. We included 5 EBPs for self-management, falls prevention, and physical activity: videoconferencing (Chronic Disease Self-Management Program, Diabetes Self-Management Program, and EnhanceFitness), telephone plus mailed materials (Chronic Pain Self-Management Program), and enhanced self-directed mailed materials (Walk With Ease). Participant and provider data included validated surveys, in-depth interviews, and open-ended survey questions. We used descriptive statistics to characterize the sample and the magnitude of change and paired *t* tests (2-tailed) and the Fisher exact test to test for change in outcomes between enrollment and 6-month follow-up. Thematic analysis was used to identify similarities and differences in outcomes within and across programs. Joint display tables facilitated the integration of quantitative and qualitative findings.

**Results:**

A total of 586 older adults, 198 providers, and 37 organizations providing EBPs participated in the evaluation. Of the 586 older adults, 289 (49.3%) provided follow-up outcome data. The mean age of the EBP participants was 65.4 (SD 12.0) years. Of the 289 EBP participants, 241 (83.4%) were female, 108 (37.3%) were people of color, 113 (39.1%) lived alone, and 99 (34.3%) were experiencing financial hardship. In addition, the participants reported a mean of 2.5 (SD 1.7) chronic conditions. Overall, the remote EBP participants showed statistically significant improvements in health, energy, sleep quality, loneliness, depressive symptoms, and technology anxiety. Qualitatively, participants shared improvements in knowledge, attitudes, and skills for healthier living; reduced their social isolation and loneliness; and gained better access to programs. Three-fourths of the providers (149/198, 75.2%) felt that effectiveness was maintained when switching from in-person to remote delivery.

**Conclusions:**

The findings suggest that participating in remote EBPs can improve health, social, and technological outcomes of interest for older adults and providers, with benefits extending to policy makers. Future policy and practice can better support remote EBP delivery as one model for health promotion, improving access for all older adults.

## Introduction

### Background

One in 6 adults living in the United States is an older adult (aged ≥60 y). This number is expected to double in the next 40 years [1]. While older adults possess the wisdom of experience and are often actively involved in taking care of themselves, their family members, and their communities, they also face a wide range of unique health challenges that come with aging [2]. A disproportionate number of older adults live with chronic health conditions: 85% have 1 chronic health condition, and 60% report managing at least two [3]. One in 4 older adults reports at least 1 fall every year, and falls remain a leading cause of death and injury among this age group [4]. These health issues—combined with social determinants of health such as older adults’ built environment, social context, and access to medical care—put older adults at risk for premature death and poorer quality of life [5]. While only accounting for 17% of the population, older adults make up 35% of health care costs, according to 2019 Medical Expenditure Panel data [6]. Furthermore, 86 cents of every dollar of health care spending goes toward chronic conditions [7,8], and the burden of chronic disease is unevenly borne by women; older adults; people of color; and people living in poverty who experience disparities in access to, and quality of, care [9].

To address these challenges, many health promotion programs have been created based on the Chronic Care Model [10], to improve access and quality of care through community-based programs that teach knowledge, skills, and self-efficacy to enhance older adults’ health and well-being in their daily lives. Among these are evidence-based programs (EBPs) that have been researched and recognized by national and federal agencies, such as the Administration for Community Living (ACL) and the Centers for Disease Control and Prevention (CDC) Arthritis Management and Well-Being Program, as being effective in promoting health outcomes through standardized interventions [11,12].

### Investigating the Effectiveness of Remote EBPs

Before the COVID-19 pandemic, many of these EBPs were primarily offered in person. This was not possible during the pandemic due to safety guidelines regarding physical distancing. At the same time, there was an increased need for programs because older adults became more isolated and less physically active, and they had less access to an overtaxed medical system [13,14]. Seeing this need, EBPs quickly pivoted to remote delivery by mail, telephone, videoconferencing, or a combination of these modes. While researchers suggest that EBPs will work as intended using different forms as long as core functions are not modified [15,16], it is unknown whether program effectiveness is maintained when switching from in-person to remote delivery. To date, there have been limited studies investigating the effectiveness of remote EBPs that were originally designed to be offered in person [17-21]. As such, we conducted a longitudinal national outcome evaluation of several EBPs to assess changes in older participants’ health.

## Methods

### Framework and Design

We used the RE-AIM (Reach, Effectiveness, Adoption, Implementation, and Maintenance) for equity framework [22] to evaluate the potential impact of remote EBPs on older adults’ health and well-being. The equity lens means that in addition to evaluating impact, we looked at outcomes across programs, sampled organizations that reach older adults who are underserved, reported EBP reach, and assessed whether there were any unintended consequences. As such, we used mixed methods to “give voice to participants as well as report statistical trends” [23]. We conducted a multisite single-group pre-post evaluation. Multiple sites were selected to facilitate the generalizability of the findings, and a single-group design was chosen to make it feasible to conduct the evaluation during the COVID-19 pandemic and due to the descriptive nature of this study.

### Ethical Considerations

This study was considered exempt from University of Washington Institutional Review Board review because the activities fell under category 2 with regard to quality improvement and program evaluation (STUDY00011549). Participants who completed the preprogram survey were given a US $10 gift card, and participants who completed the follow-up survey were given a US $20 gift card (we provided electronic gift cards unless participants requested a physical card). Providers who completed the survey received a US $10 electronic gift card.

### Selected EBPs

EBPs are health promotion programs that have been evaluated and proven to be effective. We included 5 EBPs in our evaluation: Chronic Disease Self-Management Program (CDSMP), Diabetes Self-Management Program (DSMP), EnhanceFitness (EF), Chronic Pain Self-Management (CPSMP), and Walk With Ease (WWE). These EBPs are all currently recognized by the national social services agency for older adults (ACL) and public health agencies (CDC) [11,12]. Before the COVID-19 pandemic, these programs were offered in person in group formats; they were adapted as follows for remote delivery in response to the COVID-19 pandemic: videoconferencing (CDSMP, DSMP, and EF), telephone plus mailed materials (CPSMP), and self-directed mailed materials plus enhanced support (WWE).

**Figure 1:**
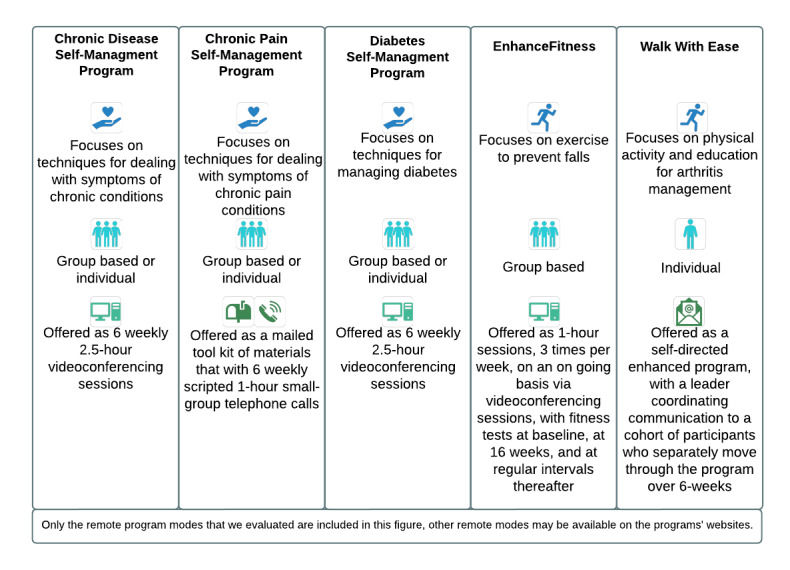
Participating remote evidence-based programs.

These 5 EBPs were selected because they had sufficient program reach and represented different remote modes (telephone, videoconferencing, mail, or a combination of these modes) and health topics (chronic disease self-management, falls prevention, and physical activity). The adaptation process largely involved planned changes to modify delivery context (eg, smaller class size; adding a cofacilitator to support engagement with, and the usability of, technology; and providing telephone options for people without access to reliable internet or videoconferencing technology) as well as some unplanned changes that occurred organically during field experience in consultation with the program leads (rather than changes to program content) [24]. It should be noted that WWE had created an enhanced self-directed program before the pandemic; we included this program in the evaluation to assess different modes of remote EBP delivery, given partners’, policy makers’, and older adults’ interest in diverse ways to engage in remote health promotion. The enhanced self-directed WWE is delivered via a cohort, with a trained leader providing web-based motivation and support to individual participants during the program period. More information about the included programs is provided below (Figure 1) and on each program’s website, including guidance for remote delivery.

### Recruitment

In early 2021, we recruited organizations delivering remote EBPs with a brief web-based interest survey and webinars through several networks: EBP training listserves, the CDC Arthritis Program and ACL grantees, the Evidence-Based Leadership Collaborative, and regional EBP networks. The organizations included social services, public health, and health care agencies that were largely community based, although some were located in clinical settings. We used maximum variation purposive sampling [25] to identify organizations of diverse sizes and types, varied provider characteristics, and from different geographic areas to aid the generalizability of the evaluation findings. This sampling prioritized organizations engaging older populations with low-income status with multiple chronic conditions who are vulnerable to both COVID-19 infection and poor health outcomes and care (eg, people of color, those with disabilities, and those living in rural areas). The eligibility criteria for organizations was the delivery of at least 1 of the 5 remote EBPs from January 2021 through March 2022.

We then used convenience sampling to invite all remote EBP participants to take part in the evaluation. Participant surveys were primarily conducted on the web using REDCap (Research Electronic Data Capture; Vanderbilt University) [26], with options to complete surveys by telephone or by mail. Participants were surveyed when they enrolled in the program and again 6 months after program enrollment (regardless of when they finished the program). At both time points, a link to the survey was emailed to participants, and they received up to 3 additional reminder emails and 3 reminder calls during the month of survey eligibility. In addition, community-based organizations encouraged baseline survey completion as part of program intake and orientation.

Furthermore, all EBP providers at participating organizations were invited to take part in a 1-time survey eliciting their perspectives regarding the impact of remote EBPs on participants as well as providers. The EBP providers included leaders (people who delivered remote programs directly) and managers (people who coordinated and oversaw program delivery).

### Data Collection

#### Quantitative

The participant outcome survey (Multimedia Appendix 1) combined several brief self-rated health measures that have been validated with older adults: single-item self-rated health [27,28], pain [29], fatigue or energy [30], loneliness (University of California Los Angeles 3-item Loneliness Scale [31]), social isolation (4-item Social Network Index [32]), depression (Patient Health Questionnaire [PHQ]-8 [33]), anxiety (Generalized Anxiety Disorder-2 [34]), physical activity (Exercise Vital Sign [35]), and social needs [36]. These cross-cutting outcomes were selected in partnership with organizations, developers or administrators, and policy makers to identify key outcomes of interest across the health promotion programs.

We also collected several program-specific outcomes [37] related to their health focus: self-efficacy for CDSMP, hypoglycemia for DSMP, and pain and the use of opioid medications for CPSMP. In addition, the preprogram survey included questions about remote EBP participant demographics: age, gender, race, ethnicity, living alone, caregiving, and chronic conditions. Rurality was defined using the participant’s zip code and several federal criteria for rural funding [38]. The 6-month follow-up survey included 3 additional quantitative items: two examining the usability of and anxiety about technology using the Senior Technology Acceptance Measure [39] and the other calculating a single-item net promoter score [40,41] to assess acceptability [42].

Provider survey data included multiple-choice questions about demographics, experience delivering EBPs, and the impact of remote EBPs on both participants and providers. Response choices were created from open-ended responses to an earlier Evidence-Based Leadership Collaborative remote EBP web-based survey in 2020 (L, Steinman, personal communication, December 2020).

#### Qualitative

The 6-month follow-up participant survey included open-ended items about program acceptability and unintended consequences or impacts. Participants who completed the follow-up survey over the telephone were also asked 2 additional qualitative questions about participating in the evaluation. Providers were also asked an open-ended question about any additional benefits for both remote EBP participants and providers.

### Data Analysis

#### Quantitative

Data were managed in REDCap [26] and analyzed using R software (R Foundation for Statistical Computing) [43]. Our primary aim was to evaluate changes in remote EBP participants’ health and well-being (self-rated health, fatigue or energy, exercise, pain, sleep, depression, anxiety, loneliness, social isolation, and program-specific outcomes) between program enrollment and 6-month follow-up. Only participants who included both baseline and 6-month follow-up data were included in these analyses. We assessed the magnitude of the changes using descriptive statistics, percentage change, and Cohen *d* effect sizes and tested for statistical significance of the changes using paired *t* tests (2-tailed). Percentage change was reported for both people who improved—or maintained—outcomes between remote EBP enrollment and 6-month follow-up, given the importance of maintaining health in an aging population [44]. Effect sizes were calculated as mean (SD) [45]. We also used descriptive statistics to characterize the sociodemographic characteristics of remote EBP participants and providers. We chose not to use the Bonferroni correction to adjust the *P* values because we carried out tests on multiple outcomes of interest across programs without a priori hypotheses (rather than 1 primary outcome of interest) [46].

#### Qualitative

Audio-recorded data were transcribed into Microsoft Word documents. These text data from transcripts and open-ended survey questions were analyzed using Dedoose software (SocioCultural Research Consultants, LLC) [47]. For qualitative data, we used thematic analysis [48,49] to understand similarities and differences across and within remote EBP acceptability and benefits or unintended consequences. Two researchers (KC and LS) created a codebook to organize important text for comparison, using deductive codes from the interview guide and inductive codes from an initial read of the transcripts [50]. We conducted 2 rounds of reliability coding, adjusted the codebook codes and definitions as needed, then completed coding using 1 coder. Coded exports were then organized in interpretation memos to summarize possible explanations for what was happening, including a summary of findings, key distinctions and counterexamples, and further points for consideration [51].

### Integration

We used joint display tables [52] and compared quantitative and qualitative results to see where the findings converged, diverged, or expanded [23].

## Results

### Participants

A total of 586 older adults participated in the evaluation over the study period, of whom 289 (49.3%) completed the 6-month follow-up survey (n=25, 8.7% completed the survey over the telephone). The primary reason for noncompletion was our inability to contact the older adults by telephone or email; only 10 (1.7%) of the 586 older adults declined to participate in the follow-up survey after being contacted.

Table 1 shows remote EBP evaluation participant demographics for those who completed both baseline and 6-month follow-up surveys. The mean age of the participants was 65.4 (SD 12.0) years. The majority were female (241/289, 83.4%) and White (184/289, 63.7%). A little more than one-third (108/289, 37.3%) identified as people of color: American Indian or Alaska Native (2/289, 0.7%), Asian (12/289, 4.2%), Black or African American (77/289, 26.6%), and Latinx (17/289, 5.9%). One in 7 (41/289, 14.2%) lived in a rural area, and 1 in 3 (99/289, 34.3%) found it “somewhat hard” or “very hard” to pay for basics such as food and housing. Of the 289 participants, 113 (39.1%) lived alone, and 58 (20.1%) were providing caregiving. The participants reported a mean of 2.5 (SD 1.7) chronic conditions, with the most common being hypertension (145/289, 50.2%), arthritis (131/289, 45.3%), diabetes (130/289, 45%), and mental health conditions (80/289, 27.7%). The prevalence of all chronic conditions is provided in Table 1, including more rare but still impactful conditions such as Crohn disease and ulcerative colitis.

**Table 1:** Demographics of remote evidence-based program evaluation participants.

Demographics		Total (n=289)		CDSMP^a^ (n=69)		CPSMP^b^ (n=47)		DSMP^c^ (n=118)		EF^d^ (n=12)		WWE^e^ (n=40)
Age (y), mean (SD)		65.4 (12.0)		60.2 (13.5)		67.5 (12.4)		64.9 (11.1)		72.2 (6.13)		71.2 (8.5)
**Gender, n (%)**												
	Female	241 (83.4)		58 (84.1)		39 (83)		93 (78.8)		12 (100)		36 (90)
	Male	48 (16.6)		11 (15.9)		8 (17)		25 (21.2)		0 (0)		4 (10)
	Nonbinary	0 (0)		0 (0)		0 (0)		0 (0)		0 (0)		0 (0)
**Race, n (%)**												
	American Indian or Alaska Native	2 (0.7)		1 (1.4)		0 (0)		1 (0.8)		0 (0)		0 (0)
	Asian	12 (4.2)		2 (2.9)		2 (4.3)		6 (5.1)		2 (16.7)		0 (0)
	Black or African American	77 (26.6)		15 (21.7)		23 (48.9)		25 (21.2)		1 (8.3)		13 (32.5)
	Native Hawaiian or Pacific Islander	0 (0)		0 (0)		0 (0)		0 (0)		0 (0)		0 (0)
	White	184 (63.7)		47 (68.1)		19 (40.4)		81 (68.6)		8 (66.7)		26 (65)
Ethnicity (Latinx), n (%)		17 (5.9)		9 (13)		2 (4.3)		5 (4.2)		1 (8.3)		0 (0)
Living in a rural area, n (%)		41 (14.2)		12 (17.4)		7 (14.9)		12 (10.2)		3 (25)		6 (15)
Somewhat hard or very hard to pay for basics, n (%)		99 (34.3)		28 (40.6)		12 (25.6)		45 (38.1)		2 (16.7)		12 (30)
Living alone, n (%)		113 (39.1)		25 (36.2)		24 (51.1)		40 (33.9)		5 (41.7)		18 (45)
Caregiver, n (%)		58 (20.1)		14 (20.3)		8 (17)		26 (22)		2 (16.7)		7 (17.5)
Chronic conditions, mean (SD)		2.5 (1.7)		2.5 (1.5)		2.6 (2.0)		2.7 (1.7)		1.7 (2.0)		1.8 (1.3)
**Chronic conditions^f^, n (%)**												
	Asthma, emphysema, chronic obstructive pulmonary disease, or chronic bronchitis	46 (15.9)	12 (17.4)		11 (23.4)		18 (15.3)		2 (16.7)		3 (7.5)	
	Arthritis (rheumatoid arthritis)	18 (6.2)	3 (4.3)		2 (4.3)		8 (6.8)		1 (8.3)		4 (10)	
	Arthritis (osteoarthritis)	72 (24.9)	18 (26.1)		6 (12.8)		28 (23.7)		3 (25)		17 (42.5)	
	Arthritis (other diagnosis)	41 (14.2)	11 (15.9)		16 (34)		8 (6.8)		0 (0)		6 (15)	
	Cancer	15 (5.2)	1 (1.4)		3 (6.4)		9 (7.6)		2 (16.7)		0 (0)	
	Diabetes	130 (45)	24 (34.8)		13 (27.7)		84 (71.2)		3 (25)		6 (15)	
	Heart trouble (eg, angina, congestive heart failure, and coronary artery disease)	49 (17)	12 (17.4)		7 (14.9)		25 (21.2)		2 (16.7)		3 (7.5)	
	Hypertension or high blood pressure	145 (50.2)	29 (42)		27 (57.4)		65 (55.1)		5 (41.7)		19 (47.5)	
	Irritable bowel syndrome	18 (6.2)	7 (10.1)		2 (5.9)		7 (5.9)		0 (0)		2 (5)	
	Kidney problems	22 (7.6)	6 (8.7)		5 (10.6)		9 (7.6)		1 (8.3)		1 (2.5)	
	Liver problems (eg, cirrhosis)	3 (1)	1 (1.4)		0 (0)		2 (1.7)		0 (0)		0 (0)	
	Mental health conditions (eg, depression, anxiety, posttraumatic stress disorder, and bipolar disorder)	80 (27.7)	28 (40.6)		15 (31.9)		31 (26.3)		0 (0)		6 (15)	
	Other digestive problems (besides irritable bowel syndrome, ulcerative colitis, and Crohn disease)	34 (11.8)	10 (14.5)		3 (6.4)		15 (12.7)		1 (8.3)		5 (12.5)	
	Stroke and other cerebrovascular disease	13 (4.5)	13 (4.3)		4 (8.5)		6 (5.1)		0 (0)		0 (0)	

^a^CDSMP: Chronic Disease Self-Management Program.

^b^CPSMP: Chronic Pain Self-Management Program.

^c^DSMP: Diabetes Self-Management Program.

^d^EF: EnhanceFitness.

^e^WWE: Walk With Ease.

^f^Less than 1% of the participants reported these chronic conditions: HIV or AIDS, Crohn disease, and ulcerative colitis.

Participants who completed the 6-month follow-up survey had similar demographic characteristics and baseline health status as those who completed only the baseline survey, with a few exceptions. A larger proportion of the CDSMP and CPSMP survey completers identified as Black compared to non–survey completers (15/69, 22% vs 13/98, 13%, and 23/47, 49% vs 31/75, 41%, respectively). WWE survey completers were less likely to be living alone (18/40, 45% vs 24/41, 59%). EF survey completers were more likely to be caregivers than not (2/12, 16% vs 0/10, 0%). Finally, across all programs except EF, survey completers were less likely to be living in a rural area than non–survey completers (37/274, 13.5% vs 109/284, 38.4%).

A total of 198 remote EBP providers (n=123, 62.1% leaders; n=75, 37.9% managers) from 107 EBP organizations in 33 states participated in the evaluation. The majority of the leaders identified as female (113/120, 94.2%). Furthermore, 4.3% (5/117) identified as Asian, 12.8% (15/117) as Black or African American, 11.7% (14/120) as Latinx, and 0.9% (1/117) as biracial. One-quarter of the leaders (31/113, 27.4%) lived in rural settings, one-third (36/120, 30%) were caregivers, and one-third (37/123, 30.1%) had ≥2 chronic conditions. One-fourth (30/121, 24.7%) identified as certified health professionals and 43.3% (52/120) as community health workers, promotoras, or other lay health providers. The leaders had a range of experience in EBP delivery: a little more than half (65/123, 52.8%) had delivered both in-person and remote programs before the survey, while 36.6% (45/123) were conducting remote EBPs for the first time. In addition to completing the survey, 26 EBP administrators, managers, and leaders took part in qualitative interviews. Most of the interview participants (22/26, 85%) identified as female and worked at community or government organizations.

### Outcomes (Quantitative)

#### Overview

**Figure 2:**
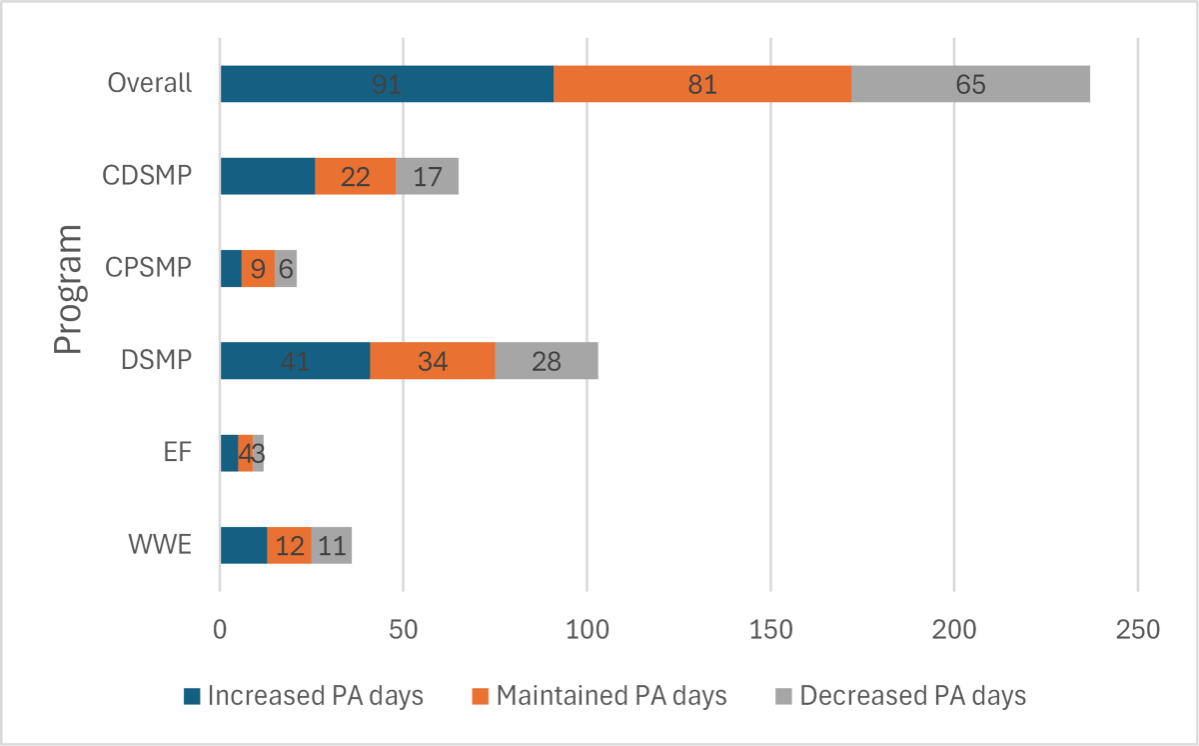
Change in the number of physical activity (PA) days between enrollment and 6-month follow-up for remote evidence-based program outcome evaluation participants. The participants were asked about the number of days on which they were physically active per week at both program enrollment and 6-month follow-up. The completion rates for this question, by program and overall, are as follows: Walk With Ease (WWE)=90% (36/40), EnhanceFitness (EF)=100% (12/12), Diabetes Self-Management Program (DSMP)=87.3% (103/118), Chronic Pain Self-Management Program (CPSMP)=45% (21/47), Chronic Disease Self-Management Program (CDSMP)=94% (65/69), and overall=82% (237/289).

Outcomes are reported by specific program and across the 5 programs included in our evaluation (Tables 2 and 3; Figure 2; Multimedia Appendix 2).

**Table 2:** Participant health outcomes at enrollment and 6-month follow-up by remote evidence-based program.

	Total (n=289), mean (SD)			CDSMP^a^ (n=69), mean (SD)			CPSMP^b^ (n=47), mean (SD)			DSMP^c^ (n=118), mean (SD)			EF^d^ (n=12), mean (SD)			WWE^e^ (n=40), mean (SD)	
Outcome	Pre^f^	Follow-up	Pre		Follow-up	Pre		Follow-up	Pre		Follow-up	Pre		Follow-up	Pre		Follow-up
Health (range 1-5) ↓^g^	3.23 (0.9)	2.80^h^ (0.9)	3.36 (0.9)		2.91^h^ (1)	3.38 (0.8)		3.21 (1.0)	3.19 (0.8)		2.71^h^ (0.8)	2.86 (1.0)		2.27 (1.0)	2.98 (0.8)		2.59^h^ (0.8)
Fatigue (range 1-10) ↓	5.49 (2.3)	4.98^h^ (2.3)	6.05 (2.1)		5.61^h^ (2.4)	5.92 (2.3)		4.88^h^ (2.1)	5.28 (2.4)		4.91 (2.5)	4.05 (1.7)		3.91 (2.2)	4.86 (2.1)		4.42 (2.1)
Pain (range 1-10) ↓	4.86 (2.6)	4.44 (2.7)	5.02 (2.4)		4.78 (2.6)	6.41 (2.3)		5.67^h^ (2.3)	4.14 (2.6)		4.16 (2.9)	4.14 (2.4)		3.64 (2.7)	4.13 (2.3)		3.68 (2.3)
Sleep quality (range 1-10) ↓	4.95 (2.5)	4.49^h^ (2.6)	5.32 (2.4)		5.07^h^ (2.8)	5.64 (2.6)		5.19 (2.5)	4.73^h^ (2.5)		4.15 (2.5)	3.91 (2.4)		3.91 (3.1)	4.14 (2.5)		3.79 (2.3)
Loneliness (range 3-9) ↓	4.67 (1.8)	4.45^h^ (1.8)	5.04 (1.9)		4.74 (2.0)	4.57 (1.7)		4.67 (2.0)	4.54 (1.8)		4.33 (1.8)	3.65 (1.0)		3.91 (1.0)	4.64 (1.7)		4.11 (1.3)
Social isolation (range 5-25) ↑^i^	15.7 (3.7)	16.2 (3.8)	15.2 (3.4)		16.3 (3.5)	15.9 (4.1)		16.7 (4.5)	15.6 (3.7)		15.6 (3.8)	18.1 (2.8)		18.6 (1.8)	16.5 (3.6)		16.8 (4.0)
Physical activity (day; range 0-7) ↑	2.3 (2.3)	2.6 (2.3)	2.0 (2.1)		2.2 (2.2)	2.5 (2.7)		2.6 (2.5)	2.2 (2.3)		2.4 (2.4)	3.3 (1.9)		3.8 (1.7)	2.7 (2.4)		3.3^g^ (2.1)
Physical activity (min; range 0-679) ↑	82.5 (113)	98.5^h^ (124)	54.7 (74)		74.7^h^ (106)	86.4 (117)		99.4 (149)	90.1 (128)		94.7 (126)	140.8 (95)		179.5^h^ (94)	107.5 (118)		130.1^h^ (118)
Depression (range 0-24) ↓	6.20 (5.3)	5.05^h^ (5.2)	7.47 (5.8)		6.07 (5.2)	7.63 (5.6)		6.50 (5.7)	5.43 (4.6)		4.83 (5.2)	4.17 (5.6)		2.10 (2.7)	3.77 (3.6)		2.77 (4.0)
Anxiety (range 0-6) ↓	1.48 (1.6)	1.30 (1.6)	1.95 (1.7)		1.65 (1.6)	1.67 (1.7)		1.54 (1.6)	1.27 (1.5)		1.26 (1.7)	0.74 (0.9)		0.60 (0.8)	1.11 (1.3)		0.67 (1.2)
Technology anxiety (range 1-10) ↓	2.74 (2.4)	2.53^h^ (2.4)	3.24 (2.7)		2.81 (2.6)	3.30 (3.1)		2.87 (2.5)	2.28 (2.1)		2.52 (2.4)	2.38 (2.7)		2.13 (2.1)	2.30 (1.8)		1.97 (1.6)
Technology usability (range 1-10) ↑	7.94 (2.6)	8.22 (2.6)	7.66 (2.5)		8.48 (2.4)	7.15 (3.3)		7.82 (2.9)	8.54 (2.3)		8.17 (2.6)	7.56 (3.0)		8.61 (2.0)	8.16 (2.4)		8.22 (2.7)
Self-efficacy (range 1-10) ↑	N/A^j^	N/A	6.16 (2.3)		6.89^h^ (2.2)	N/A		N/A	N/A		N/A	N/A		N/A	N/A		N/A
Pain interference (range 6-30) ↓	N/A	N/A	N/A		N/A	16.6 (7.3)		16.7 (7.5)	N/A		N/A	N/A		N/A	N/A		N/A
Diabetes (hypo-glycemia; range 1-7) ↓	N/A	N/A	N/A		N/A	N/A		N/A	1.92 (2.0)		1.84 (2.2)	N/A		N/A	N/A		N/A

^a^CDSMP: Chronic Disease Self-Management Program.

^b^CPSMP: Chronic Pain Self-Management Program.

^c^DSMP: Diabetes Self-Management Program.

^d^EF: EnhanceFitness.

^e^WWE: Walk With Ease.

^f^Pre: health outcomes at program enrollment; follow-up: health outcomes at 6-month follow-up from program enrollment.

^g^Lower scores indicate better health.

^h^*P*<.05 (paired 2-tailed *t* tests, except for EF, which used the Fisher exact test).

^i^Higher scores indicate better health.

^j^N/A: not applicable.

**Table 3:** Six-month effect sizes for remote evidence-based program participant health outcomes.

Outcome	Range direction	Effect size					
		Total (n=289)	CDSMP^a^ (n=69)	CPSMP^b^ (n=47)	DSMP^c^ (n=118)	EF^d^ (n=12)	WWE^e^ (n=40)
Health	1-5↓^f^	−0.37^g^	−0.37^g^	−0.23^h^	−0.44^g^	−0.58^i^	−0.35^g^
Fatigue	1-10↓	−0.23^h^	−0.20^h^	−0.42^g^	−0.13^h^	−0.25^h^	−0.33^g^
Pain	1-10↓	−0.13^h^	−0.11^h^	−0.49^g^	0.05^j^	−0.17^h^	−0.21^h^
Sleep quality	1-10↓	−0.18^h^	−0.19^h^	−0.08^j^	−0.21^h^	−0.66^i^	−0.22^h^
Loneliness	3-9↓	−0.13^h^	−0.24^h^	0.06^j^	−0.13^h^	0.58^j^	−0.30^g^
Social isolation	5-25↑^k^	0.08^j^	0.03^j^	0.07^j^	0.00^j^	0.23^h^	0.23^h^
Physical activity (days)	0-7↑	0.13^h^	0.17^h^	0.07^j^	0.10^j^	0.35^g^	0.18^h^
Physical activity (min)	3-679↑	0.15^h^	0.22^h^	0.18^h^	0.08^j^	0.36^g^	0.18^h^
Depression	0-24↓	−0.23^h^	−0.24^h^	−0.27^h^	−0.23^h^	−0.34^g^	0.06^j^
Anxiety	0-6↓	−0.08^j^	−0.12^h^	−0.36^g^	0.03^j^	0.00^j^	−0.06^j^
Technology anxiety	1-10↓	−0.13^h^	−0.17^h^	−0.23^h^	−0.08^j^	−0.03^j^	−0.24^h^
Technology usability	1-10↑	−0.05^j^	−0.06^j^	0.23^h^	−0.13^h^	0.59^i^	−0.07^j^
Self-efficacy	1-10↑	N/A^l^	0.38^g^	N/A	N/A	N/A	N/A
Pain interference	6-30↓	N/A	N/A	−0.37^g^	N/A	N/A	N/A
Diabetes (hypoglycemia)	1-7↓	N/A	N/A	N/A	−0.06^j^	N/A	N/A

^a^CDSMP: Chronic Disease Self-Management Program.

^b^CPSMP: Chronic Pain Self-Management Program.

^c^DSMP: Diabetes Self-Management Program.

^d^EF: EnhanceFitness.

^e^WWE: Walk With Ease.

^f^Lower scores indicate better health.

^g^Cohen *d* effect sizes 0.3 to 0.5=moderate.

^h^Cohen *d* effect sizes 0.1 to 0.3=small.

^i^Cohen *d* effect sizes >0.5=large.

^j^Cohen *d* effect sizes <0.1=trivial.

^k^Higher scores indicate better health.

^l^N/A: not applicable.

#### CDSMP Participants

When testing for whether these changes over time were not due to chance, CDSMP participants showed statistically significant improvements in health (mean change 0.32, 95% CI 0.117-0.530; *P*=.003; *t*_67_=3.12), energy (fatigue; mean change 0.73, 95% CI 0.061-1.408; *P*=.03; *t*_63_=2.18), sleep quality (mean change 0.89, 95% CI 0.216-1.561; *P*=.01; *t*_62_=2.64), and self-efficacy (mean change −0.53, 95% CI −0.970 to −0.091; *P*=.02; *t*_43_=-2.49). Participants with depression at baseline (19/69, 28%; PHQ-8 score <10) also significantly reduced their depression symptom severity from mean 14.7 (SD 4.2) to mean 11.5 (SD 5.1) on the PHQ-8 (*P*=.03). Overall, 58% (40/69) of the participants improved their self-efficacy over time, with a moderate effect size of 0.38. From program enrollment to 6-month follow-up, 40% (26/65) of the participants showed improvement, and 34% (22/65) of the participants maintained the number of days on which they were physically active.

#### CPSMP Participants

CPSMP participants significantly improved their energy (fatigue; mean change 1.11, 95% CI 0.248-1.968; *P*=.01; *t*_36_=2.61) and pain (mean change 0.97, 95% CI 0.186-1.763; *P*=.02; *t*_38_=2.50). Moderate effect sizes were seen for fatigue (−0.42), pain (−0.49), anxiety (−0.36), and pain interference (−0.38). The participants with pain interference at baseline (30/289, 10.4%; PROMIS [Patient-Reported Outcomes Measurement Information System] score >6) also reduced their pain interference from mean 16.9 (SD 6.6) to mean 15.5 (SD 7.3). Only 1 (2.1%) of the 47 participants was taking opioid medications at enrollment. From program enrollment to 6-month follow-up, 29% (6/21) of the participants showed improvement, and 43% (9/21) of the participants maintained the number of days on which they were physically active.

#### DSMP Participants

DSMP participants demonstrated better health (mean change 0.41, 95% CI 0.259-0.552; *P*<.001; *t*_110_=5.48). People with at least 1 hypoglycemia symptom at baseline (70/289, 24.2%) reduced their symptoms from mean 2.4 (SD 1.3) to mean 2.1 (SD 1.7); the effect size was low (−0.06). DSMP participants had a moderate effect size for overall improvement in health (−0.44). Participants with depression at baseline (19/118, 16.1%) also significantly reduced their depression symptom severity from mean 13.9 (SD 3.0) to mean 11.1 (SD 6.5) on the PHQ-8. From program enrollment to 6-month follow-up, 39.8% (41/103) of the participants showed improvement, and 33% (34/103) of the participants maintained the number of days on which they were physically active.

#### EF Participants

EF participants demonstrated better sleep quality (Fisher exact test: *P*=.001), with a large effect size of −0.66. Participants also showed strong improvements in health (effect size: −0.58) and technology usability (effect size: 0.60). From program enrollment to 6-month follow-up, 42% (5/12) of the participants showed improvement, and 33% (4/12) of the participants maintained the number of days on which they were physically active.

#### WWE Participants

WWE participants demonstrated better health (mean change 0.25, 95% CI 0.046-0.454; *P*=.02; *t*_35_=2.49), with a moderate effect size of −0.35. Participants also had moderate effect sizes for improvements in fatigue (−0.33) and loneliness (−0.30). From program enrollment to 6-month follow-up, 36% (13/36) of the participants showed improvement, and 33% (12/36) of the participants maintained the number of days on which they were physically active.

#### Across Remote EBPs

Across programs, remote EBP participants showed statistically significant improvements in their health (mean change 0.33, 95% CI 0.235-0.422; *P*<.001; *t*_267_=6.92), energy (mean change 0.56, 95% CI 0.264-0.853; *P*<.001; *t*_264_=3.73), sleep quality (mean change 0.53, 95% CI 0.245-0.812; *P*<.001; *t*_262_=3.67) loneliness (mean change 0.25, 95% CI 0.057-0.437; *P*=.01; *t*_242_=2.55), depressive symptoms (mean change 0.60, 95% CI 0.111-1.091; *P*=.02; *t*_212_=2.42), and technology anxiety (mean change 0.34, 95% CI 0.010-0.665; *P*=.04; *t*_233_=2.03). For people living with clinically significant depressive symptoms (PHQ-8 score ≥10; 24/114, 21.1% of the sample), the overall mean change in the PHQ-8 score from enrollment to 6-month follow-up was 3.025 (95% CI 1.379-4.671; *P*<.001; *t*_39_=3.717). People with clinically significant depression at baseline (52/289, 18%) also significantly reduced their depression symptom severity from mean 14.7 (SD 4.2) to mean 11.5 (SD 5.1).

The percentage change is reported in Multimedia Appendix 2. Across programs, 26.7% (66/247) to 49.8% (136/273) of the participants improved health outcomes over time, and 17.4% (41/235) to 53.8% (133/247) of the participants maintained their health. Most effect sizes (Table 3) were small (<0.3), except for change in health over time, which had an effect size of −0.37.

### Outcomes (Qualitative)

#### Participants

Overall, participants shared that they liked and enjoyed participating in the remote EBPs and identified several ways in which the program impacted their lives. First, they reported changes in their knowledge, attitudes, and practices in promoting their health and well-being. Participating in remote EBPs helped older adults manage their chronic conditions, become more active, and feel more confident and better equipped to take care of themselves:

I feel as if this program literally changed the trajectory of my life. Prior to it, I was diagnosed as prediabetic and was put on medication, which made me very sick. My next option was a very expensive diabetes drug. But, through following this program, I learned about insulin resistance and what I could do to combat my descent into diabetes. I was encouraged and coached in inspiring ways. I am now barely considered even prediabetic.

Female DSMP participant #1454; aged 66 years; living with ulcerative colitis

The program helped me understand how exercise can improve my mobility, and it encouraged me to remain active.

Male WWE participant #1346; aged 63 years; living with arthritis and asthma

Likewise, the remote EBP participants stated that they learned a lot from the program, including from the materials, leaders, and other participants. This was true across programs: WWE participants learned about new walking spots in their communities from other walkers in their cohort, while self-management program participants learned new ways and tips for managing their condition (expanded their sense of their own options) and broadened their understanding of what life was like at different severity levels of their condition. The group-based formats of the remote EBPs helped provide accountability and motivation and also provided a variety of perspectives and ideas. For some participants, the remote program helped them accept the reality and seriousness of their condition and the changes needed to manage it. In addition, participants believed that others could benefit from the program as well.

The participants also reported social benefits from being part of the programs. Many participants felt less alone, gained a sense of comfort from talking with others struggling with the same conditions, and made friends over the course of the program. A sense of camaraderie was reported often:

I liked the interaction with other people, it’s helpful to find out how others are going through. To know that there’s other people out there with a lot of pain and they’re struggling with it, made me feel not alone. Some are worse and some are better. It feels isolating a lot with pain, so that was really nice to experience, seeing others.

Female CPSMP participant #1344; aged 74 years; living alone and managing multiple chronic conditions

Furthermore, remote EBP participants from various programs who were grappling with new or existing chronic conditions, changes in mobility and function due to aging, and the challenges of physical distancing during the pandemic, emphasized the value of learning together and feeling less alone. Even participants in the remote WWE program (self-directed tool kit enhanced with a leader virtually supporting a cohort of participants) found social benefits:

I did appreciate the opportunity to meet with the group assigned to me and get the encouragement to get out and walking.

Female WWE participant #1249; aged 65 years; living with arthritis and a mental health condition

In addition, older adults shared how remote EBPs improved their access to the programs. The remote format made accessing these programs during the pandemic both safe and very convenient, in particular for people living with chronic pain or disabilities. Some participants would not have been able to participate if the programs had only been offered in person. That said, a few participants did not like the remote format and found it more difficult to access. For these participants, the downsides of not being able to meet and connect in person or the challenges with participating via telephone, mail, or videoconferencing outweighed the benefits of participating in class. Examples include issues with the technology itself (eg, poor internet connections that made the videoconferencing software freeze up) and discomfort with using technology (eg, unfamiliarity with navigating Zoom functions or unease using a mobile phone where they cannot see other participants). It should be noted that technology encompasses using a telephone (landline, smartphone, or other mobile phone) in addition to laptop computers, tablet devices, and PCs; dial-up or broadband internet; and videoconferencing platforms such as Zoom and Webex.

Finally, many participants shared that they wanted to take part in the remote EBP again, and some participants shared that they desired more follow-ups after the program ended. For these participants, there was a sense of having missed or forgotten some of what had been taught and wanting to refresh their knowledge. Others felt that they needed the motivation of continued check-ins to keep using what they had learned. In addition, some were not sure how to get their questions answered after the program ended, how to sign up for other programs offered by the organization, or whether they were allowed to take part in the program again. This points to opportunities for future supports and services after remote EBP engagement, such as monthly check-ins via telephone, videoconferencing, or social media to “keep the feelings of motivation and community after the program ends...[to] meet or discuss what folks are doing and what works and encourage each other to keep going” (female DSMP participant #1190; aged 61 years; living with multiple chronic conditions [arthritis, hypertension, diabetes, heart troubles, and a mental health condition]). Post-EBP supports and services could also provide a way to reinforce and deepen knowledge and skills that are learned and practiced during a relatively brief program and offer ways for family, friends, and caregivers to support the maintenance of program gains as well as widen program benefits to other people in the participants’ communities.

#### Providers

Table 4 summarizes provider’s perspectives on the impact of remote EBPs. Some of these impacts were expected; for example, 3 in 4 providers (149/198, 75.2%) reported improved health outcomes for older remote EBP participants, and reducing social isolation and loneliness emerged as the most common benefit for both participants and EBP providers (leaders and managers). In addition, half of the providers (102/198, 51.5%) noted that connections to other supports and services were a participant benefit; while this typically occurs during in-person EBP delivery, remote EBP delivery allowed for sharing timely and ever-changing information about testing for COVID-19 infection and recommended safety protocols, as well as referrals to services that may have paused or been shifted due to pandemic-related closures or physical distancing requirements.

**Table 4:** Perceived benefits of remote evidence-based program (EBP) delivery for participants (from providers’ perspectives) and for providers (n=198).

Benefits			Survey data, n (%)					Interview data
			Leaders (n=123)		Managers (n=75)			
**Benefits for participants (from providers’ perspectives)**								
	Improved health outcomes	93 (75.6)		56 (74.7)		Kept participants safe while also allowing them to access the benefits of these programs. The program was still effective, and the benefit it had on participants could be seen.		
	Reduced social isolation and loneliness	116 (94.3)		66 (88)		Enhanced socialization and helped with isolation, which is really needed right now. The bonds formed within the groups are really important. Clients appreciated getting checked on; many were feeling isolated during the COVID-19 pandemic.		
	Improved access to technology	52 (42.3)		30 (40)		By participating in the remote EBP, participants gained access to new or loaner technology.		
	Improved comfort with using technology	91 (74)		52 (69.3)		Increased technology literacy and comfort of participants, which encourages them to explore other web-based resources. Clients felt accomplished to have completed a class that required new technology, such as video-conferencing platforms, without help.		
	Enhanced access to other supports and services	67 (54.5)		35 (46.6)		Able to educate older adults in their program about COVID-19 vaccines. Remote program improved cross-referrals, which is good for holistically addressing health.		
	Improved access to EBPs	N/A^a^		N/A		Participants can repeat the program because it is easier to access. No concerns about driving in bad weather . Some participants liked the virtual class and want remote options in the future.		
**Benefits for providers**								
	Improved health outcomes	58 (47.2)		27 (36)		N/A		
	Reduced social isolation and loneliness	61 (49.6)		39 (52)		Able to keep working and connecting with colleagues and participants.		
	Improved access to technology	34 (27.6)		22 (29.3)		Gained access to technology via work or family, friends, and neighbors. For leaders without access, some stopped delivering the program.		

^a^N/A: not applicable (either the benefit was not one of the multiple-choice answer choices in the provider survey or did not emerge during the interview data analysis).

Some of the impacts were positive but unintended; for example, providers reported improved comfort using technology as a benefit for remote EBP participants. Increasing the usability of technology and reducing anxiety about technology are not original outcomes that in-person EBPs strived to impact, but in remote EBP delivery, opportunities arose for some participants to become more comfortable using technology (telephone, videoconferencing, or tool kits) for engaging in, and receiving, other supports and services. Comfort using technology was a more prevalent impact than access to technology, which aligns with other findings that access was a challenge even when organizations provided software or hardware [53]. Other unexpected positive impacts from the providers’ perspectives include that delivering EBPs remotely allowed them to continue providing the program to older adults throughout the COVID-19 pandemic, reach participants they had not reached before, connect participants to each other, learn how to use technology, and be able to continue working or volunteering during the pandemic.

We also gathered data via surveys and interviews on the unintended negative consequences of delivering EBPs remotely, an important aspect when evaluating the public health impact of these programs with an equity lens. For some leaders, teaching remotely was too difficult or disconnecting due to not being able to see people’s nonverbal cues and having to work harder to teach technology, engage people, or address emotional issues such that they felt that “something was lost.” Strategies for mitigating this reduced impact included reducing class sizes or duration (for both telephone and videoconferencing sessions) and requiring people attending videoconferencing sessions to have their camera on and having 2 leaders taking part so that one could focus on engagement while one managed technology.

## Discussion

### Principal Findings

Our evaluation found that remote EBP participants showed improvements on various outcomes from program enrollment to 6-month follow-up, including their self-rated health, energy, sleep quality, loneliness, depressive symptoms, and technology anxiety, within and across programs. Some program participants also reduced their anxiety, pain, pain interference, physical activity and self-efficacy, and enhanced their technology usability. The quantitative findings yielded mainly small effect sizes. This may be due to the heterogeneity of the participants; in particular, participants who did not enter the program as lonely or inactive would have no room to improve over time. The qualitative findings suggest that remote EBP participants improved knowledge, attitudes, and skills on how to live healthier lives; reduced social isolation and loneliness; and gained better access to programs. In addition, providers shared that they too benefited from delivering programs remotely by staying connected, having access to technology, and improving their own health and well-being.

In some cases, the findings support previous research on the effectiveness of the remote EBPs that were part of our evaluation; for example, 1 study of 213 videoconferencing CDSMP participants in rural and remote Ontario, Canada, found similar improvements in self-rated health, energy, and psychological well-being (a measure related to depressive symptoms) 4 months after their last class [54]. Another study of 97 telephone plus tool kit CPSMP participants in Cleveland, Ohio, United States, also reported better pain outcomes immediately after program completion [17]. Furthermore, a study of self-directed WWE participants (n=270) in rural and urban North Carolina, United States, who were living with arthritis found that participants also reduced fatigue at follow-up 1 year after program enrollment [21]. Our findings are also comparable to those of similar health promotion programs, such as increased physical activity and reduced depression for a remote DSMP [55].

In other cases, our findings contrast with those of previous research; for instance, 1 study of remote-delivered EF [20] found that the participants (n=15) decreased their knee pain as measured by the Knee Injury and Osteoarthritis Outcome Score; however, all study participants had symptomatic knee osteoarthritis, and the postprogram outcome was measured directly after active intervention at 12 weeks. It may be that our evaluation participants did not significantly improve pain outcomes due to their less severe baseline pain. In addition, the study of CPSMP participants mentioned previously [17] found statistically significant improvements in sleep and depression; our evaluation participants too improved their sleep quality and reduced their depressive symptom severity, but this change was not statistically significant. Our differing findings may be due to our smaller sample size (47 vs 97), which lowered our power to detect significant change, or a longer follow-up period (6 months vs 6 weeks), during which improvements may have been attenuated.

Furthermore, it should be noted that our outcome evaluation was not designed to compare in-person EBP delivery with remote EBP delivery. We used a common set of outcome measures of interest to older adults, EBP delivery organizations, and policy makers; as such, some of these measures look at similar constructs as the in-person EBP effectiveness studies but use different instruments, and some of these measures that are newly being evaluated (eg, loneliness and depression) were not evaluated in the in-person EBP research studies. In addition, we used a different time period compared to studies that evaluated the effectiveness of in-person EBP delivery: we looked at changes between program enrollment and 6-month follow-up, regardless of program duration, whereas prior effectiveness research looked at pre-post change over time based on program duration [21,56-59]. Our sample sizes were also smaller than those of previous in-person EBP research studies; therefore, the lack of statistical significance may be due to a lack of power to detect change. That said, it may be helpful to contextualize our remote EBP outcome evaluation findings with those from in-person EBP outcome evaluations. Remote CDSMP evaluation participants reported improvements in self-efficacy, health, energy (fatigue), sleep quality, physical activity, and depression, all outcomes that were also reported in previous studies of in-person CDSMP [37,60-62]. Furthermore, remote CDSMP evaluation participants did not show the significant improvements in pain outcomes that were seen in research with in-person program participants. Remote CPSMP evaluation participants reported outcome improvements similar to those of in-person CPSMP participants regarding pain and pain interference [56,63], while our evaluation added evidence for remote CPSMP participant improvements in energy (fatigue), anxiety, and physical activity; these 3 outcomes were not assessed in in-person CPSMP studies. However, remote CPSMP participants did not report significant improvements in self-efficacy, as was reported in previous in-person CPSMP studies. Looking at the DSMP, both remote and in-person delivery participants showed reduced hypoglycemia symptoms, health, and depression [57]. Earlier research with in-person program participants found improved self-efficacy as well, and remote program participants showed improvement in terms of their physical activity days. For EF, both remote and in-person program participants reported improvements in self-rated health as well as physical activity [64,65]. This evaluation adds novel findings on improved sleep quality and the usability of technology for remote program participants. In addition, in-person EF participants have reported reduced depression in other studies [64]. Looking at WWE, both in-person and remote program participants have demonstrated improvements in health, fatigue, and physical activity [21]. Remote WWE participants reduced their loneliness, which was not assessed in previous studies of in-person WWE [21]. Previous research of in-person WWE also found participant improvement in pain and self-efficacy [21].

Although EBPs shifting to remote delivery was necessitated by the pandemic-related lockdown and other restrictions [24], the findings suggest that remote delivery can improve access to quality health promotion programs outside the COVID-19 pandemic context; for example, a caregiver for a person living with dementia can join a physical activity program from home to support their own health while not having to find respite care for their loved one, an older adult living with chronic pain can join a class even if they are experiencing elevated pain levels that would make it hard to leave even their bedroom, and a program leader can deliver the program across a region in Spanish to participants who may not have access to language-specific health promotion. This is similar to other studies of remote EBPs (eg, the value for people living with cancer to be able to participate from a distance despite living with weakened immune systems [66]).

Adapting the format and channels through which an EBP is delivered [67] is considered an appropriate modifiable intervention characteristic to better align with the needs and preferences of participants, as well as organizational and geographic contexts in which a remote option improves program feasibility and sustainability [68]. Implementation science increasingly recognizes the need for adaptations [69] to improve the intervention-context fit (eg, increasing both practical and value fit [70]; increasing EBP feasibility to a given context [71]; and making sure that the EBPs can be delivered for different systems, organizations, providers, and participants than was originally studied [72]), support people living with multiple chronic conditions because comorbidity is the rule rather than the exception [73], and ensure EBP effectiveness by evaluating the intervention in different settings with varying provider and participant attributes [74]. Our complementary process evaluation found that delivering in-person EBPs remotely did not require modifications to core program components [53], suggesting that fidelity to the active ingredients of the program models was maintained. That said, some of the observed lack of improvement in health outcomes by remote EBP participants may be due to failures in implementation (eg, inadequate intervention dose due to technology issues) that negatively impacted program effectiveness [42].

### Strengths and Limitations

Our evaluation comes with several strengths. First, we conducted a pragmatic evaluation across the country in partnership with policy makers and delivery organizations so that the findings would have direct implications for improving older adults’ health equity. Specifically, we built upon trusting relationships and took time to foster new relationships to engage people most impacted by implementing remote EBPs; we also gathered qualitative data to put quantitative outcomes in context for future quality improvement [75]. Second, we included multiple brief, validated health outcomes across various EBPs and diverse participants and providers, centering outcomes that are important and meaningful to participants, providers, organizations, and policy makers that were not measured in previous studies [76]. These include social factors such as social isolation and loneliness and mental health factors such as depression and anxiety, which lead to premature mortality for older adults [77,78]. While measuring multiple outcomes across heterogeneous populations and programs made it harder to see large effect sizes, it better reflects how organizations work (delivering multiple programs) and how participants view health more holistically rather than as just 1 primary outcome. It may also be that small effect sizes suggest that these programs provided primary or secondary prevention to delay the onset of more impairing symptoms and conditions that was not picked up from our measures or relatively study time frame [79]. Third, collecting qualitative as well as quantitative data from the perspectives of both participants and providers provides consistent measures to compare across studies as well as stories and unanticipated outcomes to explore more broadly in future research.

Looking at limitations, first, using a single-group design with the lack of a comparison group or randomization means that we cannot attribute change in health outcomes over time to participating in the remote EBP. Second, we recognize that the lack of statistical significance when assessing for whether change was due to chance or program participation may be due to small sample sizes that lack power. This is partly why we collected qualitative data from both providers and participants: we wanted to understand the magnitude of change from their perspectives. Third, our response rate was lower than is typical in controlled research studies, with only half of the remote EBP participants (289/586, 49.3%) providing follow-up data. This was due to pandemic-related logistical and methodological challenges faced by our evaluation team, program providers, and older adult participants; conducting an evaluation during the COVID-19 pandemic has been deemed more challenging than conducting evaluations in conflict areas [80]. This suggests limitations to both internal validity through biases such as selection bias (eg, persons who liked the program or had access to a mobile phone or the internet to complete the evaluation were overrepresented) and external validity, with the COVID-19 pandemic being a unique context, because of which our findings may not be generalizable (eg, were improvements in social connection due to the high level of disconnectedness faced by everyone during the pandemic-related lockdown?). Finally, our convenience sample only included group-based programs and as such may not be applicable to one-on-one EBPs. We also did not have access to data on the entire remote EBP delivery population; therefore, we cannot comment on the representativeness of our outcome evaluation sample. Our process evaluation [24] suggests that persons who have been historically underserved by EBPs (eg, people of color, those with disabilities, and those living in rural areas) can be reached through remote delivery; however, care must be taken to ensure that remote delivery does not widen the health inequities caused by the digital divide.

### Conclusions

In closing, the findings from this outcome evaluation suggest that participating in remote EBPs can improve health, social, and technology outcomes of interest for older adults, providers, and policy makers (Textbox 1). Future policy and practice can better support remote EBPs, improving access for all older adults (regardless of income, geography, and ability) and strengthening delivery organizations.

**Lessons learned for evidence-based program (EBP) administrators, developers, and implementers.**

**Key takeaways**

- Delivering EBPs remotely (by telephone, videoconferencing, mail, or a combination of these modes) offers a new format for engaging older adults in quality health promotion programming.
- Outcome evaluation findings suggest that remote EBPs are effective at improving health, social, and technology outcomes for older adult participants and providers who deliver the programs, although impacts are not experienced universally across programs or outcomes.
- Interviews and surveys with remote EBP participants and providers suggest that these outcomes can be achieved because remote EBPs are acceptable; increase knowledge, skills, motivation, support, and accountability; connect people to peers and leaders; and support tech access and comfort.
- Include brief, validated pre- and postprogram surveys in your routine program delivery to understand changes in outcomes that matter to your community, organization, and funders. (The surveys may include traditional health outcomes you assessed with in-person EBPs as well as new outcomes, such as social isolation, loneliness, technology anxiety, and technology usability. Our survey measures are cited in this paper and available on request.)
- Gather data on remote EBP participant demographics to understand who is being reached and who is not being reached by remote programs to strategize engagement via other outreach strategies or in-person EBP modes.
- Remote EBP delivery may improve access to health promotion programming for people facing inequitable access to in-person programming; however, caution is needed to ensure that remote delivery does not widen the digital divide; for example, while remote delivery can support rural-dwelling older adults from areas without nearby programs or dependable transportation, the lack of reliable internet in some rural areas may necessitate a telephone or mail remote EBP delivery mode.
- Supports for remote EBPs include orienting participants to using program technology before and during the program and providing ways for program participants to connect after the program ends.

## Appendix

Multimedia Appendix 1 Participant outcomes survey.

Multimedia Appendix 2 The percentages of improvement and maintenance in participant health outcomes at enrollment and 6-month follow-up in remote evidence-based programs for health promotion.

## Abbreviations

ACL: Administration for Community Living
CDC: Centers for Disease Control and Prevention
CDSMP: Chronic Disease Self-Management Program
CPSMP: Chronic Pain Self-Management Program
DSMP: Diabetes Self-Management Program
EBP: evidence-based program
EF: EnhanceFitness
PHQ: Patient Health Questionnaire
PROMIS: Patient-Reported Outcomes Measurement Information System
RE-AIM: Reach, Effectiveness, Adoption, Implementation, and Maintenance
REDCap: Research Electronic Data Capture
WWE: Walk With Ease
