# Supplementary material for: Remote Evidence-Based Programs for Health Promotion to Support Older Adults During the COVID-19 Pandemic and Beyond: Mixed Methods Outcome Evaluation
Source: JMIR Aging. 2024 Jun 13;7:e52069. doi: 10.2196/52069 (PMC11211707; doi:10.2196/52069)

Program Survey

Thank you for participating in our program.

Before you start the program, we would like to learn a little bit about you and your health. Please complete the following survey before your first program.

I have two quick things to tell you about the survey:

1. First, you are welcome to skip any questions you do not want to answer, and you can stop the survey at any time.

2. Second, all of your responses are confidential, which means we will never share your responses with your program leader or anyone else. Of course, you may share your information with your program leader and other participants in your workshop if you choose.

Thank you for taking the time to fill out the survey! We really appreciate it.

ABOUT YOU

We would like to know a little about you so we know who we are reaching.

Who is completing this survey? (select one)

- I am a program participant completing my own survey
- I am staff completing the survey on behalf of a program participant

Participant Name: ___________________ **Participant Phone number**: _______________

Email address:______________________ **Participant ZIP code:** _______________

Today's date:__________

1. What is your age? _____

2. Do you live alone? (circle one): Yes / No

3. Are you a caregiver? (circle one): Yes / No

4. What is your gender? (circle one): Male Female Transgender Other: _______

5. What is your ethnicity? (circle one): Hispanic/Latino Not Hispanic/Latino

6. What is your race (circle all that apply):

White/Caucasian Black/African-American Native Hawaiian/Pacific Islander

Asian American Indian/Native American Other:______________________

7. What health (chronic) conditions are you living with? (check all that apply)

- □ I have no health conditions
- □ Asthma, emphysema, COPD, or chronic bronchitis
- □ Cancer
- □ COVID-19 long-haul
- □ Depression, anxiety, PTSD, bipolar or other mental health condition
- □ Diabetes
- □ Heart trouble (e.g. angina, congestive heart failure, or coronary artery disease)
- □ Hypertension or high blood pressure
- □ HIV or AIDS
- □ Kidney problems
- □ Liver Problems (such as cirrhosis)
- □ Stroke or other cerebrovascular disease

Arthritis

- □ Rheumatoid (RA)
- □ Osteoarthritis (OA)
- □ Other arthritis

Digestive problems

- □ Crohn's disease
- □ Irritable bowel syndrome (IBD)
- □ Ulcerative colitis
- □ Other digestive problems

8. How hard is it for you to pay for the very basics like food, housing, heating, medical care, and medications? (circle one):

Not hard at all Somewhat hard Very hard Declined N/A

9. How many years did you attend school (circle one):

0 1 2 3 4 5 6 7 8 9 10 11 12 13 14 15 16 17 18 19 20 21 22

10. Which program are you currently taking? (check one)

- ☐ Chronic Disease Self-Management Program
- ☐ Chronic Pain Self-Management Program
- ☐ Diabetes Self-Management Program
- ☐ EnhanceFitness
- ☐ Healthy IDEAS
- ☐ HomeMeds via phone
- ☐ Walk with Ease
- ☐ Other: write-in: ___________________________

11. How is your program being delivered? (check one)

- ☐ Live group online using Zoom or other video-conferencing
- ☐ Mailed materials + phone calls
- ☐ Self-directed toolkit
- ☐ Other ____

Program Survey

We would like to know how you are doing. Please answer the questions below. You may skip any questions you do not wish to answer.

HEALTH

In general would you say your health is: …………………………………………… (circle one)

1	2	3	4	5
Excellent	Very good	Good	Fair	Poor

FATIGUE (tiredness)

We are interested in learning whether or not you are affected by FATIGUE. Please circle the number below that describes your fatigue or tiredness **in the past week**:

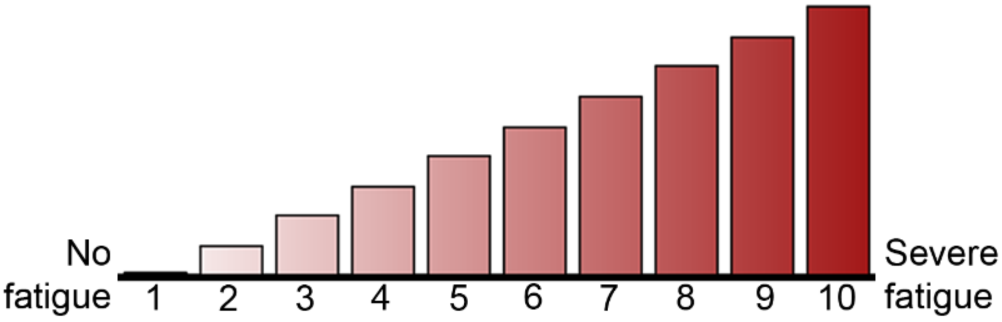

PAIN

We are interested in learning whether or not you are affected by PAIN. Please circle the number below that describes your level of pain **in the past week**:

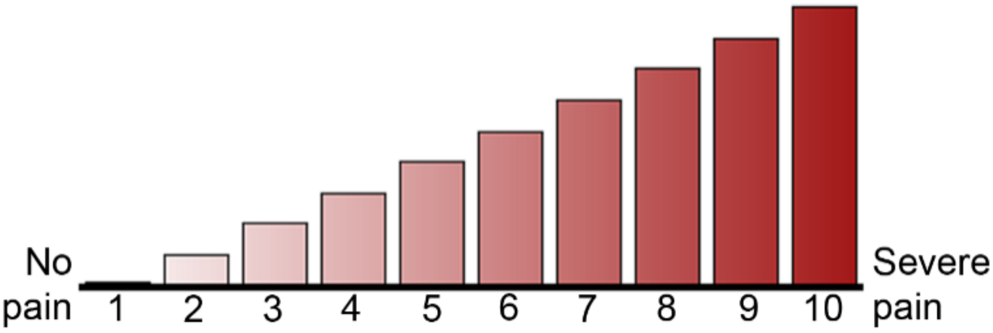

SLEEP

We are interested in learning whether or not your SLEEP has been affected. Think about how many hours you slept, how easy it was to fall asleep, how often you woke at night or earlier than expected, how refreshing your sleep was. Please circle the number below that describes your SLEEP QUALITY **in the past week**:

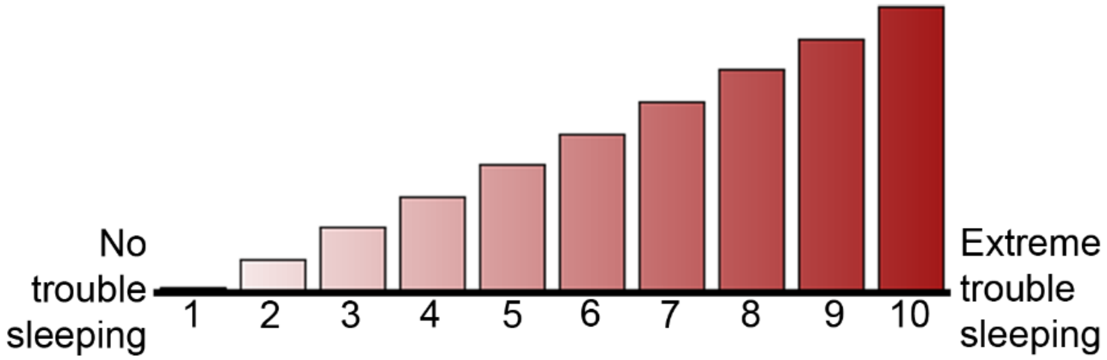

SOCIAL CONNECTIONS

We would like to know about your social connections and how you feel about them.

**How often do you feel...**	**Hardly ever**	**Some of the Time**	**Often**
1. That you lack companionship?	1	2	3
2. Left out?	1	2	3
3. Isolated from others?	1	2	3

**In a typical week, how often do you…**	**Never**	**Once a week**	**2 days a week**	**3-5 days a week**	**Nearly every day**
4. See family, friends or neighbors in-person? This can include in-person visits with social distancing.	1	2	3	4	5
5. Talk with family, friends or neighbors by phone or video chat?	1	2	3	4	5
6. Use email, text messaging, or internet to communicate with family, friends or neighbors?	1	2	3	4	5
7. Attend religious services (e.g. church, temple, mosque) online, by phone, or in-person?	1	2	3	4	5
8. Attend classes or other groups you belong to online, by phone, or in-person?	1	2	3	4	5

DIFFICULT EMOTIONS

Over the **past 2 weeks,** how often have you been bothered by any of the following problems?
(circle one number on each line)

	None at all	Several days	More than half the days	Nearly every day
1. Feeling nervous, anxious or on edge	0	1	2	3
2. Not being able to stop or control worrying	0	1	2	3
3. Little interest or pleasure in doing things	0	1	2	3
4. Feeling down, depressed or hopeless?	0	1	2	3
5. Trouble falling asleep, or staying asleep, or sleeping too much?	0	1	2	3
6. Feeling tired or having little energy?	0	1	2	3
7. Poor appetite or overeating	0	1	2	3
8. Feeling bad about yourself, or that you are a failure or have let your family down	0	1	2	3
9. Trouble concentrating on things, such as reading the newspaper or watching television	0	1	2	3
10. Moving or speaking so slowly that other people could have noticed. Or the opposite – being so fidgety or restless that you have been moving around a lot more than usual.	0	1	2	3

If you checked of any of the above, how difficult have those problems made it for you to do your work, take care of things at home, or get along with other people? (*circle one*)

1	2	3	4
Not difficult at all	Somewhat difficult	Very difficult	Extremely difficult

PHYSICAL ACTIVITIES

These questions are about **aerobic** (endurance) **physical activity**. This is any activity that causes you to breathe a little harder or to raise your pulse. Examples of activities include walking, running, dancing, swimming, bicycling, etc. Stretching is not an aerobic activity.

1. In the past week, how many **days** did you do aerobic activity? (*circle one*)

0 days	1 days	2 days	3 days	4 days	5 days	6 days	7 days

2. In the past week, how many **minutes per day** did you do these aerobic activities? (*circle one*)

less than 15 minutes	15-29 minutes	30-44 minutes	45-59 minutes	60-74 minutes	75-90 minutes	More than 90 minutes

ACCESS TO RESOURCES

We understand it can be difficult to access resources during this time. Which basic needs are hard for you to meet at this time? (*check all that apply*)

- ◻ I do not need help meeting any basic needs
- ◻ Transportation
- ◻ Housing
- ◻ Financial
- ◻ Safety
- ◻ Caregiving help
- ◻ Health care
- ◻ Medications
- ◻ Social support
- ◻ Food (e.g. groceries, meals)
- ◻ Services in my language COVID-19 resources (e.g., info, testing, vaccines)
- ◻ Other: ____________

USE AND COMFORT WITH TECHNOLOGY

"Technology" includes any tools to participate in a program from your home, such as the internet, phone, computer, camera, speakers or tablet.

1. What technology do you **use** to participate in this program?

	I own this	I borrow this from someone in my household	I borrow this from a community resource (senior center, public library)
a. A computer (desktop or laptop)?			
b. A tablet (i.e., iPad)?			
c. A smart phone (i.e., phone with a video screen and access to internet)			
d. Other type of phone (e.g. landline or cell phone without internet or screen)			
e. Speaker and microphone (audio) that allows you to talk to and hear people?			
f. A camera (video) that allows you to see people and they see you			
g. Other (please specify)			

2. Does family, a friend or a neighbor help you with your technology to participate in this program? This could include setting up, logging on or using your technology.

- ☐ Yes
- ☐ No

3. <u>Please circle the number</u> below to rate **how nervous or worried you feel about using whatever technology you used to participate in the program such as phone, computer, or tablet**:

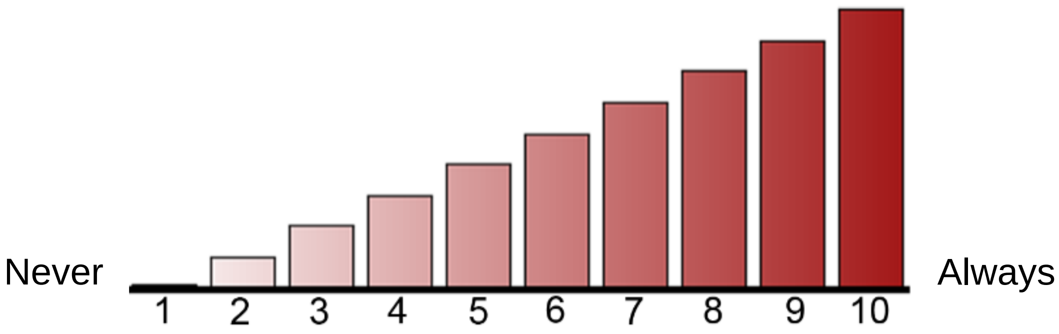

4. <u>Please circle the number</u> below to rate **whether you have the skills to use whatever technology you used to participate in the program such as phone, computer, or tablet**:

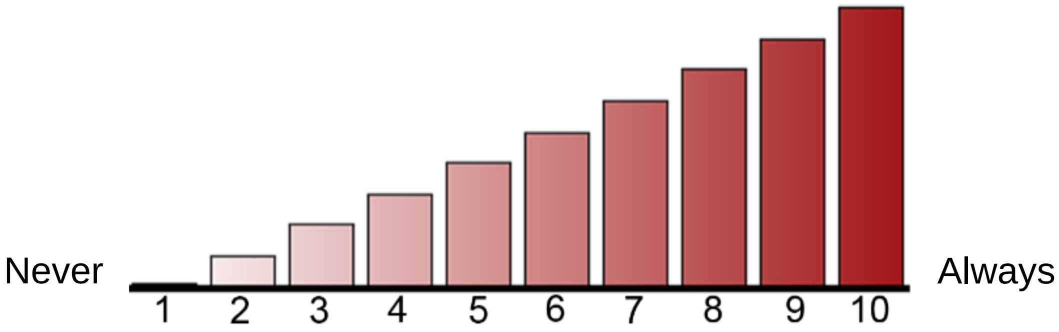

Supplement: Multimedia Appendix 1 [file aging_v7i1e52069_app1.pdf]
