# Supplementary material for: Remote Evidence-Based Programs for Health Promotion to Support Older Adults During the COVID-19 Pandemic and Beyond: Mixed Methods Outcome Evaluation
Source: JMIR Aging. 2024 Jun 13;7:e52069. doi: 10.2196/52069 (PMC11211707; doi:10.2196/52069)
Supplement: Multimedia Appendix 2 [file aging_v7i1e52069_app2.docx]

**Supplementary Table 1. Percent improvement and maintenance in participant health outcomes at enrollment (pre) and 6-month follow-up, by remote EBP. ^a^**

| **Improved**  ***Maintained*** |  | **Overall** | **CDSMP** | **CPSMP** | **DSMP** | **EF** | **WWE** |
| --- | --- | --- | --- | --- | --- | --- | --- |
| **Outcome** | **Range**  **Direction ^b^** |  |  |  |  |  |  |
|  |  |  |  |  |  |  |  |
| Health | 1-5  ↓ | 107 (38.8%)  *138 (50.0%)* | 20 (30.3%)  *40 (60.6%)* | 14 (31.8%)  *23 (52.3%)* | 53 (46.5%)  *48 (42.1%)* | 5 (41.7%)  *7 (58.3%)* | 15 (38.5%)  *20 (51.3%)* |
| Fatigue | 1-10  ↓ | 136 (49.8%)  *59 (21.6%)* | 34 (52.3%)  *10 (15.4%)* | 28 (65.1%)  *8 (18.6%)* | 49 (43.0%)  *26 (22.8%)* | 6 (50.0%)  *3 (25.0%)* | 19 (48.7%)  *12 (30.8%)* |
| Pain | 1-10  ↓ | 118 (43.1%)  *77 (28.1%)* | 23 (35.9%)  *19 (29.7%)* | 30 (66.7%)  *5 (11.1%)* | 39 (34.2%)  *41 (36.0%)* | 6 (50.0%)  *2 (16.7%)* | 20 (51.3%)  *10 (25.6%)* |
| Sleep quality | 1-10  ↓ | 118 (43.1%)  *77 (28.1%)* | 33 (50.8%)  *10 (15.4%)* | 20 (47.6%)  *7 (16.7%)* | 49 (43.0%)  *26 (22.8%)* | 7 (58.3%)  4 (33.3%) | 3 (59.0%)  *7 (17.9%)* |
| Loneliness | 3-9  ↓ | 132 (48.4%)  *54 (19.8%)* | 24 (39.3%)  *24 (39.3%)* | 8 (22.2%)  *20 (55.6%)* | 32 (32.0%)  *47 (47.0%)* | 1 (2.9%)  *5 (45.4%)* | 10 (27.0%)  *22 (59.4%)* |
| Social isolation | 5-25  ↑ | 105 (44.4%)  *41 (17.4%)* | 30 (46.9%)  *11 (17.2%)* | 9 (50.0%)  *2 (11.1%)* | 44 (40.7%)  *22 (20.4%)* | 5 (45.4%)  *2 (18.2%)* | 17 (45.9%)  *4 (10.8%)* |
| Physical activity days | 0-7  ↑ | 91 (38.6%)  *82 (34.7%)* | 26 (40.0%)  *22 (33.8%)* | 6 (28.6%)  *10 (47.6%)* | 41 (39.8%)  *34 (33.0%)* | 5 (45.4%)  *4 (36.4%)* | 13 (36.1%)  *12 (33.3%)* |
| Physical activity minutes | 0-679  ↑ | 98 (41.7%)  59 (25.1%) | 29 (43.9%)  *17 (25.8%)* | 9 (42.9%)  *6 (28.6%)* | 38 (37.3%)  *25 (24.5%)* | 5 (45.4%)  *4 (36.4%)* | 17 (48.6%)  *7 (20.0%)* |
| Depression | 0-24  ↓ | 127 (47.2%)  *64 (23.7%)* | 31 (50.0%)  *12 (37.5%)* | 21 (53.8%)  *8 (20.5%)* | 56 (48.3%)  *27 (23.3%)* | 4 (33.3%)  *5 (41.7%)* | 15 (37.5%)  *12 (30.0%)* |
| Anxiety | 0-6  ↓ | 66 (26.7%)  *133 (53.8%)* | 17 (28.3%)  *27 (45.0%)* | 14 (38.9%)  *20 (55.6%)* | 26 (24.8%)  *58 (55.2%)* | 1 (10.0%)  7 (70.0%) | 8 (22.2%)  *21 (58.3%)* |
| Tech anxiety | 1-10  ↓ | 70 (30.2%)  *109 (47.0%)* | 20 (33.3%)  *60 (40.0%)* | 8 (40.0%)  *8 (40.0%)* | 28 (26.2%)  *56 (52.3%)* | 5 (45.4%)  *5 (45.4%)* | 9 (27.3%)  16 (48.5%) |
| Tech usability | 1-10  ↑ | 98 (42.4%)  *68 (29.4%)* | 16 (59.3%)  *27 (45.8%)* | 8 (40.0%)  *8 (40.0%)* | 29 (27.4%)  *42 (39.6%)* | 5 (45.4%)  *6 (54.5%)* | 9 (25.7%)  *16 (45.7%)* |
| Self-efficacy | 1-10  ↑ | -- | -- | 29 (58.0%)  *4 (8.0%)* | -- | -- | -- |
| Pain interference | 6-30  ↓ | -- | -- | -- | -- | -- | 8 (50.0%)  *3 (18.8%)* |
| Diabetes  (hypoglycemia) | 1-7  ↓ | -- | -- | -- | 29 (30.5%)  *45 (47.4%)* | -- | -- |

^a^ Percent improvement = #(%) of participants who improved on each outcome measure.

^b^ The arrows in the second column indicate whether higher (↑) or lower (↓) scores indicate better health.
